# Neutrophil extracellular traps and acute kidney injury: a narrative review

**DOI:** 10.3389/fimmu.2026.1853708

**Published:** 2026-07-13

**Authors:** Shuyuan Zhou, Jiawei Lu, Jie Zheng, Yunqiang Liao, Si Chen, Haizhen Wu, Jiahui Xiong, Xiaosheng Li, Yang Tang

**Affiliations:** 1 First Clinical Medical College of Gannan Medical University, Ganzhou, China; 2 Department of Nephrology, The First Affiliated Hospital of Gannan Medical University, Ganzhou, China; 3 Department of Traditional Chinese Medicine, The First Affiliated Hospital of Gannan Medical University, Ganzhou, Jiangxi, China

**Keywords:** acute kidney injury, mechanisms, neutrophil extracellular traps, tissue damage, treatment

## Abstract

Neutrophil extracellular traps (NETs) are complex, network-like structures comprising chromatin filaments bound to various antimicrobial proteins. While they play a role in host defence under physiological conditions, there is evidence to suggest that excessive production or impaired clearance of NETs may contribute to the pathological progression of acute kidney injury (AKI). This review analyses the heterogeneous roles of NETs composed of various components, such as histones, DNA and associated enzymes, in mediating renal tissue damage. It also explores their involvement in ischaemia-reperfusion injury, haemolytic uraemic syndrome, sepsis and autoimmune-associated AKI. Furthermore, this article reviews existing NET intervention strategies and considers the potential for personalised treatment strategies in different disease contexts. However, translating NET-related research into clinical practice faces numerous challenges, including the lack of standardised biomarkers for real-time monitoring of NET levels and preserving the host’s essential anti-infective immune pathways while inhibiting tissue damage. Future research should therefore focus on elucidating the precise regulatory mechanisms underlying NET formation, as well as exploring methods for the targeted delivery of drugs to the kidneys. This would enhance the specificity and safety of treatment.

## 1 Introduction

AKI is a serious and highly challenging clinical problem, with an incidence rate of over 50% among patients in intensive care units (1, 2). Furthermore, large-scale multinational studies indicate that approximately 5.7% of critically ill patients require renal replacement therapy, and the mortality rate associated with this group is as high as 60.3% (3). From an economic and long-term prognostic perspective, in high-income countries alone, AKI results in annual losses of approximately US$1 billion and claims 300,000 lives, whilst contributing to 170,000 patients progressing to end-stage renal disease and 300,000 patients advancing to advanced chronic kidney disease each year (4). Despite advances in medical care, the incidence of AKI continues to rise, and in-hospital mortality rates in ICU settings have long remained at high levels of 40% to 80%; its poor prognosis has not improved significantly over the past few decades (5, 6). AKI is triggered by a variety of complex pathologies, including ischaemia, toxicity and infection; its common pathophysiological mechanism manifests as substantial damage to renal tubular epithelial and endothelial cells, as well as severe impairment of microvascular function (5, 7, 8). Neutrophils, as the key immune cells that are the first to respond within 24 hours of injury, play a vital role in this process (9–11).

Among the effector cells of the innate immune system, neutrophils are the most abundant (12, 13). They possess a wide range of potent antimicrobial substances; however, as these substances can also damage human tissue, their activity is strictly regulated by three mechanisms: phagocytosis, degranulation and the release of NETs (14). NETs are a network-like structure composed of a modified chromatin scaffold and various antimicrobial proteins (15); they were discovered by Brinkmann et al. (16) when neutrophils were stimulated with phorbol 12-myristate 13-acetate (PMA). In the early stages of infection, NETs can establish a local defence barrier by trapping pathogens, releasing antimicrobial proteins and limiting microbial spread; conversely, they can also reduce tissue damage through mechanisms that regulate pathogen size (14, 17). However, if they are produced in excess or are not cleared in a timely manner, they may lead to a number of diseases, such as systemic lupus erythematosus (SLE), rheumatoid arthritis (RA), ANCA-associated vasculitis and arthritis (18). NETs also play a pivotal role in kidney-related diseases (19); they are implicated in conditions such as renal ischaemia-reperfusion injury(RIRI) (20), haemolytic uraemic syndrome (21) and systemic lupus erythematosus (22), including AKI (23). NETs primarily cause damage to renal tissue through direct cytotoxicity, amplification of inflammatory cascades, and the mediation of damage to distant organs (24); this damage can lead to the development of AKI, manifested as an acute rise in serum creatinine levels or a reduction in urine output over a fixed period (25). This review will examine the role of NETs in AKI and explore potential treatment approaches, thereby offering new insights into the diagnosis and management of AKI (23).

### 1.1 An overview of NETs

Neutrophils are the most abundant cells of the innate immune system in peripheral blood; they originate in the bone marrow and are able to rapidly reach the site of infection through chemotaxis, adhesion and endothelial migration. They eliminate pathogens by phagocytosis and the release of reactive oxygen species(ROS) and cathepsins, whilst simultaneously secreting various mediators to regulate the immune response (26, 27). NETs are specialised structures released by neutrophils in response to infection and inflammation to enhance their antibacterial properties (16); they are structured around chromatin fibres composed of DNA and modified core histones, and selectively enrich various granule proteins including neutrophil elastase (NE), myeloperoxidase (MPO), lactoferrin, cathepsin G, eukocyte proteinase 3 (PR3), lysozyme C, and azurophilin, as well as cytoplasmic proteins such as calmodulin-binding proteins S100A8/A9 and S100A12, catalases, glycosidases, and cytoskeletal proteins (28). Among these, histones account for the highest proportion, at approximately 70 per cent, whilst NE is the most abundant non-histone protein; calpetin (29) is the primary antifungal effector molecule. However, this composition is not static. Research by Dwyer (30) et al. shows that although NETs possess a highly conserved ‘core signature’, their protein profiles differ significantly under different stimuli, such as between non-mucous and mucous Pseudomonas aeruginosa strains, with a total of 45–80 proteins identified, of which only approximately 33 constitute a common core; the mucoid strain PA581 possesses 22 unique proteins, whilst the clinical strain 2192 possesses 39 unique proteins. Whether these differences translate into functional differences was not directly demonstrated in the original study and requires further investigation.

In terms of function, NETs are capable of trapping, neutralising and killing bacteria (31), fungi (32), viruses (33) and parasites (34). In particular, when pathogens are too large to be effectively phagocytosed, neutrophils tend to release NETs to exert extracellular control, as is the case with Candida albicans hyphae (35). NETs primarily achieve a certain degree of extracellular bactericidal activity against Gram-negative and Gram-positive bacteria under specific conditions by physically trapping bacteria, degrading virulence factors and releasing high concentrations of antimicrobial components; they represent an innate immune mechanism independent of phagocytosis (16). However, recent studies have revealed that, under the persistent influence of recurrent inflammation and pathogenic factors, neutrophils—unable to effectively resolve the issue—lead to the excessive release or abnormal accumulation of NETs. These abnormal NETs may cause excessive damage to host tissues through mechanisms such as histotoxicity, immune activation and thrombosis, or prevent damaged tissues from being effectively repaired, and may even trigger abnormal immune responses (36). NETs are therefore also considered to be a potential aetiological factor in many diseases.

### 1.2 The formation and mechanisms of NETs

**Figure 1:**
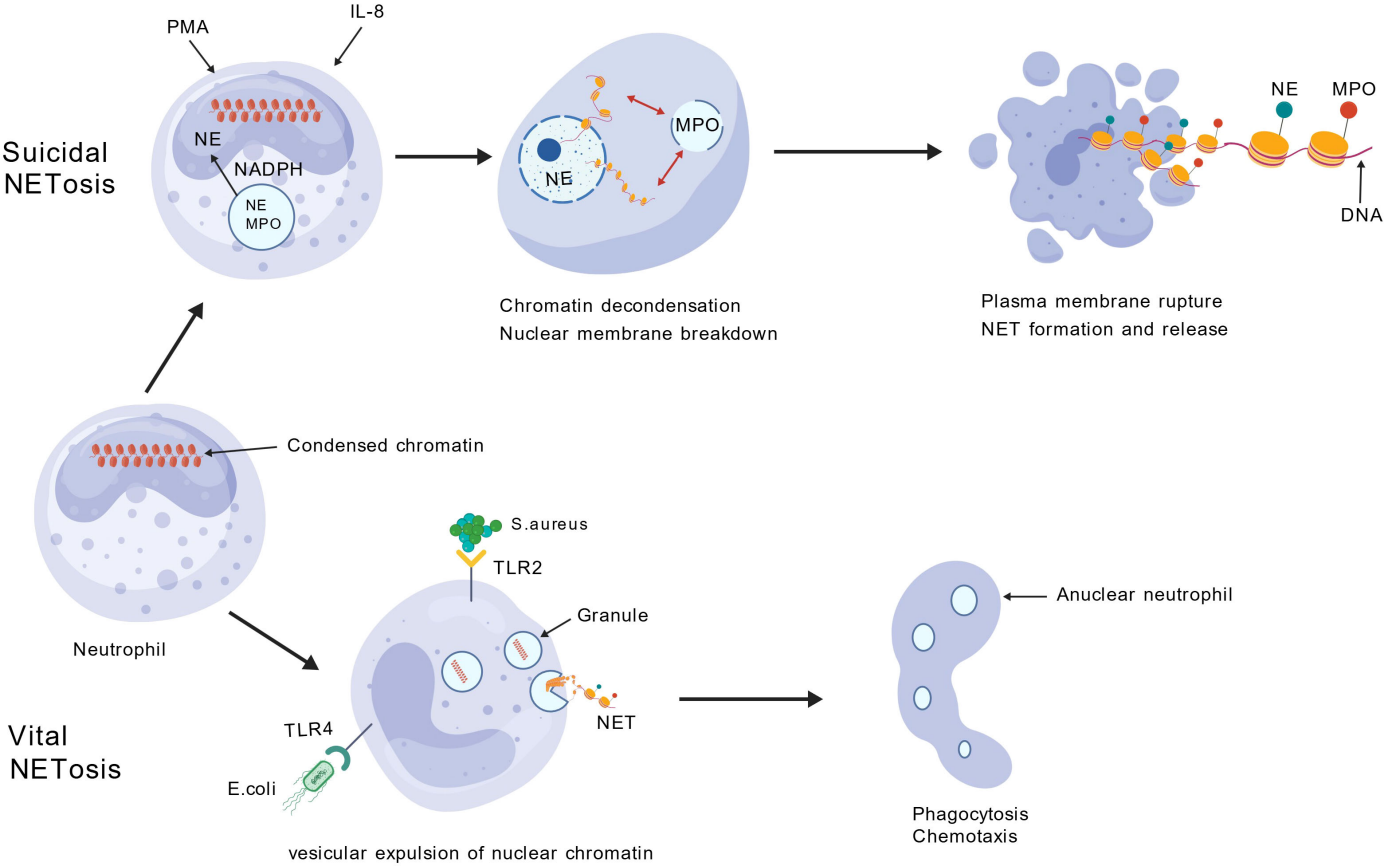
NETosis release pathways. Suicidal NETosis: When stimuli such as pathogens or inflammatory factors bind to receptors on the surface of neutrophils, they trigger a cascade of reactions leading to chromatin disassembly and the rupture of the nuclear and granule membranes, allowing chromatin to come into contact with granule proteins in the cytoplasm to form NETs. Ultimately, the cell membrane ruptures, releasing the NETs. Vital NETosis: Under specific conditions, neutrophils may undergo ‘non-suicidal’ NETosis. When neutrophils are activated, intracellular calcium ion concentrations rise, activating PAD4, which leads to chromatin disassembly and the formation of NETs. These are released in the form of vesicles; during this process, the neutrophils do not die immediately [Created with BioGDP.com (41)].

Neutrophils initiate NET formation (NETosis) via two distinct pathways (37–40), dictated by the nature of the stimulus (**Figure 1**) (42, 43). Classical “suicidal” NETosis, typically triggered by stimuli such as lipopolysaccharide(LPS), IL-8, or PMA, is characteristically NADPH oxidase 2(NOX2)-dependent (44). In this pathway, NOX-derived ROS promote the translocation of MPO and NE from granules into the nucleus (45, 46). Within the nucleus, these enzymes degrade chromatin, while peptidyl arginine deiminase 4 (PAD4) facilitates decondensation via histone H3 citrullination (47–49). This sequential ROS-MPO-NE-PAD4 axis ultimately culminates in the rupture of the nuclear and plasma membranes, releasing NETs as the cell undergoes programmed death (16, 37, 50).

However, in response to signals from acute infections mediated by certain pathogens, such as Staphylococcus aureus (51) and Candida, or by ‘activated’ platelets (52), neutrophils can rapidly release NETs within a shorter time window of 5–60 minutes. Unlike the suicidal model, this pathway is NOX2-independent and driven by extracellular calcium influx (53). The calcium-activated potassium channel SK3 acts as a key mediator, and its activation alone is sufficient to induce this process. Rising intracellular Ca^2+^ levels activate PAD4 to promote chromatin decondensation. This genetic material and the granule proteins are then packaged into microvesicles and expelled from the cell; during this process, the cell surface membrane reseals to maintain its integrity, leaving behind an anuclear neutrophil that remains viable, motile and capable of continuing to perform its immune functions (54).

**Figure 2:**
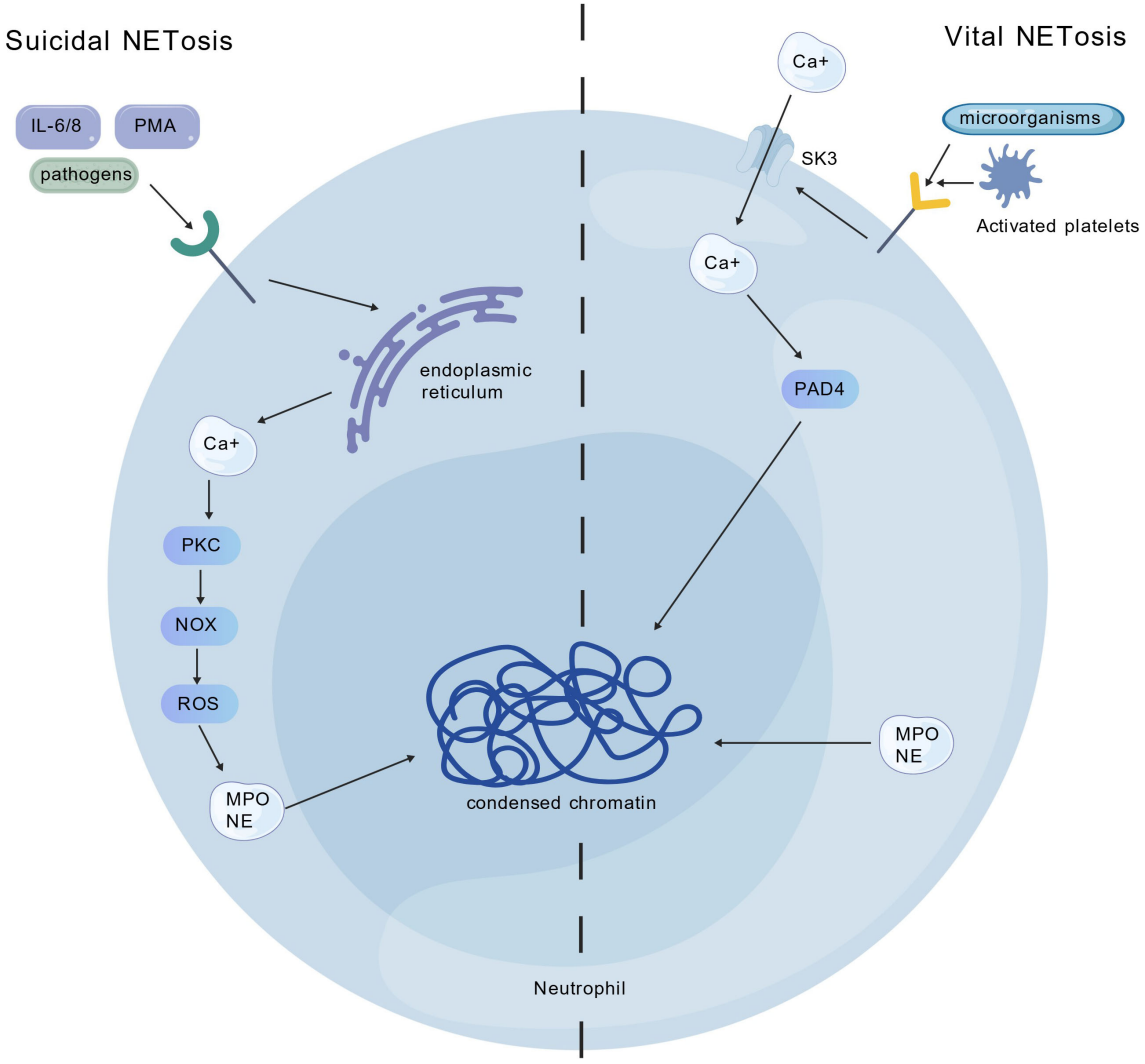
Intracellular signalling pathways of NETosis. Suicidal NETosis: Upon activation of neutrophils, calcium ions are released, and protein kinase C (PKC) is activated. Subsequently, ROS levels are elevated by activated NOX, whilst NOX also promotes the degradation of cytoplasmic granules containing MPO and NE. Cytoplasmic PAD4, together with NE and MPO, induces citrullination of H3, leading to chromatin decondensation. Ultimately, NETs are formed and released into the extracellular space. Vital NETosis: Following neutrophil activation, calcium ions are transported into the neutrophil via SK3. The rise in intracellular calcium concentration activates PAD4, which catalyses the conversion of histone arginine to citrulline, leading to chromatin uncoiling and the formation of NETs. These are released in the form of vesicles; during this process, the neutrophils do not die immediately [Created with BioGDP.com (41)].

In summary, depending on the triggering factors and cellular outcomes, the release of NETs can provisionally be classified into suicidal NETosis and vital NETosis (55) (**Table 1**). Both of these pathways for NET formation result in the release of NETs, but their mechanisms differ (**Figure 2**).

**Table 1:** Comparison of the two forms of NETosis.

Features	Suicidal NETosis	Vital NETosis
Trigger	Microorganisms (bacteria/fungi) (56, 57), Endogenous complement (58), PMA (59), Urate crystals (60), etc.	Ionophores: A23187 (calcium), nigericin (potassium) (42, 53), Activated platelets (52)
key enzyme	NOX2/MPO (46)/NE	PAD4 (61, 62)
Cell membrane integrity	Eventually, they rupture completely, releasing their contents (63)	NETs may be released via vesicles, whilst part of the cell membrane remains intact (38)
Types of histone modifications	NE-cleaved histone (63, 64)	PAD4-mediated citrullination (cit-H3)
Function and pathological significance	Defence against acute infections, but overactivation leads to tissue damage (e.g. sepsis)	Chronic inflammation, autoimmune diseases (such as SLE), thrombosis

Furthermore, it is generally believed that the scaffold components of NETs originate from uncoiled nuclear DNA; however, research by Yousefi (65) et al. has shown that neutrophils pre-treated with granulocyte-macrophage colony-stimulating factor (GM-CSF) and subsequently subjected to brief stimulation with C5a or LPS are capable of releasing mtDNA into the extracellular space to form a network-like structure within a rapid time window, without undergoing cell death. Unlike lytic cell death caused by the release of nuclear DNA, neutrophils that release mtDNA do not exhibit apoptotic features such as DNA fragmentation or phosphatidylserine exposure; their cell membranes remain intact, and they even survive longer than resting neutrophils. However, the primary mechanism underlying this release has not yet been clearly elucidated. Some studies (66) suggest that mitochondrial functional integrity serves as a key upstream regulatory node in the formation of calcium-mediated, NOX-independent NETs; however, no definitive experimental evidence has yet been provided regarding the specific mechanism, and further investigation is required. Lood (67) et al. have demonstrated that mitochondrial ROS are key drivers of NET formation, particularly in the spontaneous NETosis of low-density granulocytes (LDGs) in autoimmune diseases such as SLE; this further underscores the crucial role played by mitochondria in NETs.

### 1.3 The mechanism of damage to NETs

**Figure 3:**
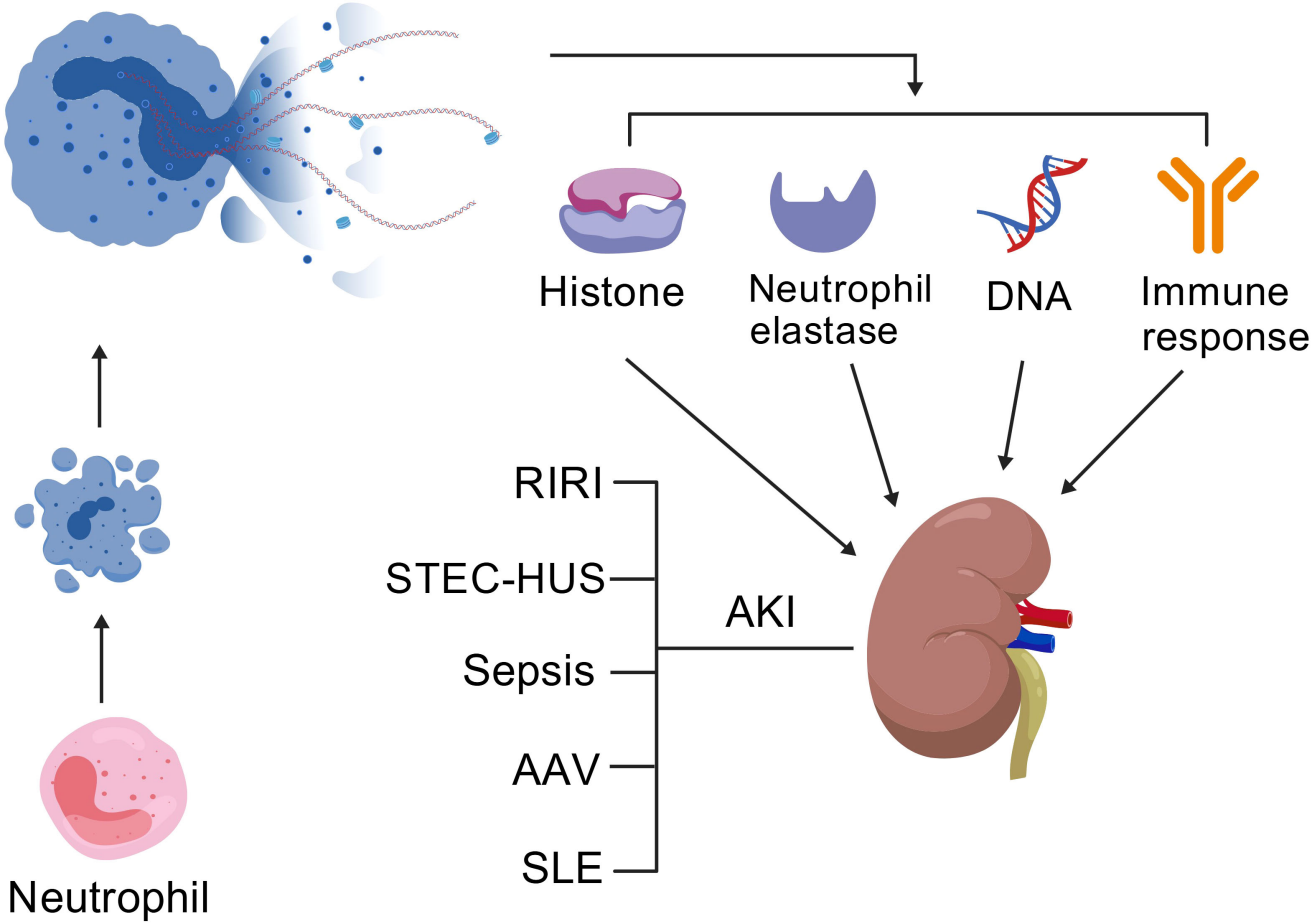
NETs and kidney-related diseases: NETs cause kidney damage of various aetiologies through mechanisms such as histones and DNA, NE, autoimmune reactions, and excessive activation of the complement system [Created with BioGDP.com (41)].

Although NETs play a vital role in anti-infective and immune defence mechanisms by capturing and killing pathogens and limiting the spread of infection, their excessive release or dysregulation can lead to pathological consequences such as tissue damage, exacerbated inflammatory responses, the induction of autoimmune diseases, and the promotion of thrombosis. Among kidney-related diseases, NETs induce inflammatory responses, tissue fibrosis and immune damage through mechanisms such as histones and DNA, NE, autoimmune reactions and excessive activation of the complement system, making NETs a major aetiological factor in kidney-related diseases (68) (**Figure 3**).

#### 1.3.1 Neutrophil elastase

NE is a potent serine protease (69), primarily stored in the azurophilic granules of neutrophils. Upon infection, neutrophils release NE through degranulation following activation. NE can degrade various bacterial proteins, such as virulence factors and surface proteins, thereby directly killing pathogens; it also plays a role in the activation or inactivation of cytokines and chemokines, influencing the course of the inflammatory response. During NET formation, the NE plays a crucial role: it enters the neutrophil nucleus and specifically cleaves or degrades core histones, particularly H4 (70), directly triggering extensive chromatin decondensation. Subsequently, MPO binds to the chromatin and acts in synergy with the NE to further enhance decondensation; however, this synergy does not depend on the peroxidase activity of MPO (71). This process causes the originally densely packed chromatin to depolymerise and relax, resulting in the DNA being ‘unbound’ from the nucleosomes, where it is tightly wrapped around a core of histones. Furthermore, the NE cleaves and activates the pro-inflammatory protein Gasdermin D, thereby forming pores in the plasma membrane, which ultimately leads to the leakage of cellular contents into the extracellular space, such as a mixture of DNA strands and antimicrobial substances, including the NE and MPO (72). However, research by Pieterse et al. suggests that when the NET load exceeds the limited endocytic capacity of microvascular glomerular endothelial cells via the RAGE-clathrin pathway, the elastase carried by the persistently exposed NETs hydrolyses the core protein of the endothelial cell adhesion junctions, VE-cadherin (73), rapidly disrupting intercellular adhesion junctions, increasing endothelial barrier permeability and enhancing the transendothelial flux of albumin (74). As the junctions disintegrate, β-catenin is released from the junctions into the nucleus, leading to upregulation of fibrogenic genes such as Snail1, MMP9 and α-SMA, thereby inducing endothelial-mesenchymal transition, which in turn accelerates the progression of renal fibrosis (74, 75) and may even contribute to the development of renal failure.

#### 1.3.2 DNA

If NE acts as a trigger, then DNA filaments serve as the ‘physical cornerstone’ of the entire NETs damage mechanism. As a fundamental structural component of NETs, they consist of a network of filaments formed from chromatin released by cells, which, under specific conditions, contains mitochondrial DNA. As research into them deepens, they are no longer regarded as mere remnants of dead cells, but rather as an ‘alarm signal’ resulting from oxidative modification. As a three-dimensional network structure, it provides binding sites for various proteases, such as NE, MPO and histones; this aggregation leads to the accumulation of high concentrations of proteases and autoantigens in the extracellular space, thereby promoting vascular lesions and the activation of inflammasomes (67, 76–78). At the same time, the vast three-dimensional physical scaffold formed by these DNA strands also traps red blood cells and platelets (79) circulating within the microvasculature; these trapped platelets then release platelet factor 4 (PF4) (80) and RANTES (81) PF4 binds to the DNA matrix, making the NET structure more compact, whilst conferring strong resistance to DNase degradation and inducing more neutrophils to produce NETs (82), thereby leading to thrombus formation (83), and even multi-organ failure and death. Secondly, the DNA within NETs plays a key role in inducing the production of type I interferon (IFN); once taken up by target cells—human peripheral blood mononuclear cells (PBMCs) and the monocyte cell line (THP1)—it can induce the expression of IFN-β and other pro-inflammatory cytokines via the cGAS-STING signalling pathway (84), thereby causing damage to the glomerular filtration barrier, clinically manifesting as proteinuria, or even inducing lupus nephritis(LN), which may ultimately lead to renal failure (67). Furthermore, DNA can also be recognised by pattern recognition receptors (PRRs) in the body as damage-associated molecular patterns (DAMPs) (85, 86), inducing the massive production of pro-inflammatory cytokines such as tumour necrosis factor (TNFα) and interleukin (IL-6), thereby exacerbating the inflammatory environment in the kidneys (87).

#### 1.3.3 Histone

Building upon the physical framework provided by DNA, histones act as the primary effector molecules responsible for delivering the lethal blow. As part of the innate immune system, they possess broad-spectrum antimicrobial capabilities and are effective against bacteria, fungi, viruses and pathogens residing within drug-resistant biofilms (88, 89). However, this cytotoxic effect is not specific and can also damage host cells (90). Consequently, histones are also thought to be associated with the exacerbation of damage in a variety of conditions, such as sepsis (90), severe trauma, small-vessel vasculitis, and microvascular complications (91) involving the liver (92), brain and lungs (93). The kidneys are no exception (94), as histones can directly damage glomerular endothelial cells, podocytes and mesangial cells, leading to glomerular capillary necrosis and the development of proteinuria (95). This direct cytotoxicity may be mediated through two distinct mechanisms. One involves histones—particularly H3 and H4, which are rich in positively charged arginine and lysine residues—binding to negatively charged phospholipids on the cell membrane, such as phosphatidylserine. This disrupts the stability of the lipid bilayer and creates pores, leading to cell lysis; this mechanism primarily affects renal tubular epithelial cells and microvascular endothelial cells. The other mechanism involves the substance entering the cell via existing ion channels or by creating new pores, causing an influx of calcium ions. This leads to cell membrane depolarisation, increased endoplasmic reticulum pressure and cell death (96). Secondly, histones can also act as DAMPs by binding to and activating Toll-like receptors (TLRs, primarily TLR2 and TLR4) on endothelial cells and macrophages, thereby triggering a local inflammatory response. This leads to the release of pro-inflammatory cytokines such as TNF-α, IL-6 and IL-8, as well as further neutrophil infiltration, creating a vicious cycle that exacerbates tubular damage and dysfunction (20, 94, 95). During this process, not only do histamines directly induce platelet activation and aggregation (97), but the TNF-α produced also accelerates and enhances fibrin formation and the density of its network by inducing procoagulant activity in endothelial cells (particularly via TF) (98). The presence of fibrin, in turn, stimulates the vigorous proliferation of parietal epithelial cells lining the Bowman’s capsule; these proliferating cells, together with infiltrating macrophages, accumulate within Bowman’s space, forming a crescent-like structure (i.e. a crescent) (99). Crescents compress the normal glomerular capillary tuft, causing it to collapse and harden, ultimately leading to the loss of function of the entire nephron.

#### 1.3.4 The immune and complement systems

As the final link in this chain, the activation of the complement system transforms local NET-induced damage into a systemic immunopathological process. NETs consist primarily of double-stranded DNA, which carries a negative charge on its surface; the spherical head of C1q can recognise and bind to this, tightly enveloping the fibrillar structure of the NETs and thereby initiating the cascade reaction of the classical pathway (100–104). During this process, C4 and C3 are cleaved, producing fragments such as C4b and C3b (105), which deposit on the NETs. C3d (a degradation product of C3b) binds to complement receptor 2 (CD21) on the surface of B cells; this binding significantly lowers the activation threshold for B cells, causing them to begin producing autoantibodies against DNA and histones (106). As the cascade progresses, C5a (anaphylatoxin) is also produced; as one of the most potent pro-inflammatory factors generated by the complement cascade (107), it causes local vasodilation and increased vascular permeability, leading to the leakage of plasma proteins and inflammatory cells into the interstitial spaces, resulting in redness, swelling and exudative damage. It also attracts neutrophils from the bloodstream to accumulate at sites where NETs are present (such as glomeruli and blood vessel walls), where they release lysosomal enzymes, proteases and ROS, and generate further NETs (77, 106, 108). Interestingly, C1q can also inhibit DNase-I, thereby preventing the degradation of NETs, which in turn attracts more C1q and leads to stronger complement activation (106). This process results in the continuous production and activation of complement within the patient’s body, leading to the massive ‘depletion’ of complement proteins in the bloodstream (109). This is consistent with the clinically observed significantly low levels of C3 and C4 in the serum of SLE patients during active disease. In AAV, NETs can also generate C5a by activating the complement alternative pathway, inducing the release of further NETs and creating a vicious cycle; simultaneously, histones and MPO within the NETs directly damage endothelial cells, leading to oligoimmunological crescentic nephritis (110).

## 2 NETs and acute kidney injury

AKI is a clinical syndrome characterised by a rapid decline or loss of renal function (25), manifested by an increase in serum creatinine (SCr) of ≥0.3 mg/dL within 48 hours, or an increase in SCr to ≥1.5 times the baseline value within 7 days, or urine output <0.5 mL/kg/h persisting for 6 hours, and is classified into stages 1–3 according to severity (111). Although the diagnostic criteria are clear, the aetiology of AKI is complex and diverse, with pathophysiological mechanisms often intertwined. Studies have shown that NETs are involved in the pathogenesis of AKI caused by RIRI, haemolytic uraemic syndrome, and immune-related diseases (such as systemic lupus erythematosus and anti-neutrophil cytoplasmic antibody-associated vasculitis) (14, 112, 113).

### 2.1 NETs and RIRI

RIRI, a common cause of AKI (114), involves a complex cascade of events initiated by the interruption and subsequent restoration of renal blood flow or the restoration of blood flow following ischaemia. These mechanisms include oxidative stress, inflammatory responses, and various forms of cell death (115), such as apoptosis, necrosis, autophagy, and programmed cell death. During renal ischaemia-reperfusion, renal tubular epithelial cells exhibit high sensitivity to hypoxia, potentially leading to extensive cell necrosis and the release of intracellular contents into the extracellular space. These substances are known as DAMPs, which include molecules released by damaged cells (HMGB1). HMGB1 binds to pattern recognition receptors (PRRs) on the surface of neutrophils (87), such as RAGE, TLR2, TLR4, TLR9, etc.

Current mechanistic studies suggest that following this binding, intracellular PAD4 is activated to perform ‘protein deimination,’ converting positively charged arginine on H3 into uncharged citrulline (Cit-H3) (116, 117). In some cellular models, PAD4 has also been observed to induce the translocation of the pro-inflammatory transcription factor NF-κB (p65) from the cytoplasm to the nucleus, potentially initiating the synthesis of the chemokine MIP-2—a potent recruiter of neutrophils (118). During this process, the unravelling of chromatin (decondensation) occurs as the binding strength between histones and DNA decreases.

Once chromatin is fully unraveled, neutrophil membranes may rupture, ejecting decondensed DNA fibers and granule proteins—a process forming NETs. Histological analyses in murine models demonstrate that NETs accumulate in the corticomedullary region following ischemia/reperfusion, where they colocalize with neutrophils and activated platelets (83, 112). It is hypothesized that this extracellular DNA also acts as an activation signal, binding to PRRs (such as TLRs) on platelets to induce the release of P-selectin and PF4/CXCL4, which further promotes NET extrusion (112). While this suggests a potential positive feedback loop exacerbating renal injury, the extent to which this occurs in human clinical AKI remains an area of active investigation. In these models, P-selectin binds to its ligand PSGL-1 to promote platelet-neutrophil adhesion, while PF4 enhances neutrophil adherence to the vascular endothelium (119–121).

Furthermore, substances such as Cit-H3 and NE are released alongside DNA, which not only exert direct toxic effects on adjacent renal tubular epithelial cells, disrupting cell membrane integrity and leading to tubular cell necrosis, thereby exacerbating microcirculatory impairment, but also trigger inflammatory cascade reactions by activating the NF-κB signalling pathway, further recruiting and activating neutrophils, thus creating a vicious cycle (20, 122). However, it remains a point of clinical debate whether NETs are the primary etiological drivers of injury or secondary amplifiers of an existing inflammatory milieu. This distinction is critical for the development of NET-targeted therapies, as the translational relevance of these vicious cycles from animal models to human patients requires further validation.

### 2.2 NETs and haemolytic uraemic syndrome

Shiga toxin-producing Escherichia coli (STEC)-associated haemolytic uraemic syndrome (STEC-HUS) is a clinical syndrome caused by Shiga toxin, characterised by haemolytic anaemia, thrombocytopenia and AKI; it is a major cause of acute infection in children (123). NETs play a crucial dual role in its pathogenesis: on the one hand, NETs participate in innate immune defence by trapping and clearing pathogens; on the other hand, the ability to degrade NETs is significantly reduced in patients with STEC-HUS, which may lead to abnormal accumulation of NETs, thereby further exacerbating damage to the renal tubules and glomeruli through mechanisms such as pro-inflammatory, pro-coagulant and complement activation (21). STEC colonises the intestine and releases Shiga toxin 2 (Stx2), which crosses the intestinal epithelial barrier to enter the bloodstream. In the bloodstream, Stx2 binds to polymorphonuclear neutrophils (PMNs) and triggers NETosis via an NOX-dependent pathway (124); however, there remains controversy regarding whether PMNs possess the characteristic Gb3 receptor on their surface (125). Interestingly, Landoni et al. (126) argue that Stx2 itself does not directly trigger NETosis in neutrophils, but rather renders platelets extremely sensitive to inflammatory signals. These highly activated platelets act like ‘glue’, capturing neutrophils in the bloodstream via P-selectin to form platelet-neutrophil mixed aggregates (PMN-Plts Aggregates), thereby inducing NETosis. This discrepancy may be related to the use of sub-threshold doses of non-NETosis-inducing agents and the washing of treated platelets prior to the addition of neutrophils to remove free toxins.

In summary, as NETosis occurs, glomerular endothelial cells (HGECs)—the primary site of damage in HUS—are not only directly injured by NETs but are also continuously stimulated by them as “danger signals”. Once activated, HGECs begin to secrete large amounts of pro-inflammatory cytokines: IL-8 (a chemokine that attracts more PMNs) and IL-6 (a systemic inflammatory regulator), thereby activating a local inflammatory response and indirectly exacerbating glomerular damage. Persistent granule protein release, a cytokine storm, and microthrombi ultimately lead to loss of glomerular filtration function, triggering acute renal failure (113).

### 2.3 NETs and sepsis

AKI is a common complication of sepsis and is associated with an extremely high mortality rate (127–129). Sepsis-associated acute kidney injury (S-AKI) is typically defined as AKI meeting the KDIGO criteria within 7 days of the onset of sepsis (as defined by the Sepsis-3 criteria) (130). As a life-threatening critical condition, sepsis is characterised by a dysregulated immune response to infection, which involves a series of complex, cross-systemic abnormalities (131, 132); S-AKI is no exception.

Neutrophils are normally the immune system’s ‘first line of defence’, but in the complex environment of sepsis, their numbers, lifespan, migratory behaviour and killing mechanisms undergo pathological changes, transforming them into the ‘masterminds’ behind organ damage and immunosuppression (133, 134). In healthy individuals, neutrophils express almost no CCR2 receptors on their surface; they rely primarily on CXCR2 receptors to navigate to sites of infection (135). During sepsis, bacteria and their products (such as the endotoxin LPS or lipoteichoic acid LTA) enter the bloodstream. These substances act as ligands, activating Toll-like receptors (TLR2, TLR4, TLR9) on the surface of circulating neutrophils, leading to a significant decrease in CXCR2 expression (136, 137). Furthermore, through the MyD88-activated NF-κB signalling pathway, this induces neutrophils to begin synthesising and expressing CCR2 receptors in large quantities (138). This ‘receptor switching’ endows neutrophils with a chemotactic ability that is not inherent to them—namely, the ability to respond to CCR2 ligands (such as CCL2). Furthermore, the systemic inflammation caused by sepsis leads to the production of large quantities of chemokines, particularly CCL2 (also known as MCP-1), in distal tissues such as the kidneys (134, 138). As circulating neutrophils are now equipped with CCR2 receptors, they are able to detect the high concentrations of CCL2 released by the kidneys, causing them to divert from the primary site of infection and instead be drawn towards the renal CCL2 signal. Furthermore, the kidneys also release high levels of inflammatory cytokines, such as IL-8, attracting large numbers of neutrophils to accumulate in the renal microvasculature and interstitium (130).

Another important characteristic of neutrophils is the formation of NETs. While NETs serve as a barrier of the innate immune system to effectively localise infection, substantial evidence suggests that their over-production may contribute to pathology by mediating immune thrombogenesis and direct cytotoxicity, thereby potentially driving systemic inflammatory response syndrome and multi-organ dysfunction (139). NETs are rich in granule proteins and histones, which, once released into the extracellular space, may function as potent DAMPs. It is hypothesized that these components bind to TLR2 and TLR4 receptors on the surface of renal tubular cells, initiating downstream signalling cascades that may promote the synthesis and release of pro-inflammatory cytokines. This process is linked to increased oxidative stress, ROS production, and mitochondrial damage in proximal tubule epithelial cells, ultimately manifesting as tubular vacuolisation, microvascular congestion, and reduced glomerular filtration (24, 140–142). Furthermore, it is proposed that neutrophils and NETs may damage the endothelial glycocalyx and increase endothelial permeability (143); for instance, components such as elastase and matrix metalloproteinases (MMPs) may cleave VE-cadherin, disrupting endothelial junction integrity (73). Additionally, citrullinated Cit-H3, a core NET component, has been linked to significant pro-inflammatory and pro-permeability properties. It likely induces the formation of actin stress fibres and endothelial cell contraction, widening intercellular gaps and causing plasma leakage, which leads to interstitial oedema and increases the diffusion distance for oxygen to reach tubular epithelial cells (144). Concurrently, NETs deposited on the vascular wall may trap platelets, inducing widespread microvascular obstruction via immunothrombosis and resulting in insufficient renal perfusion, thereby potentially exacerbating AKI (145). It is important to acknowledge that the pathophysiology of NETs in AKI remains a subject of active investigation. While NETs are frequently identified as biomarkers of disease severity, their precise contribution as either causal mediators or secondary amplifiers of injury in the context of S-AKI is still being disentangled. In a prospective follow-up study of 136 patients with S-AKI, He et al (146). observed abnormally elevated plasma NET levels in non-survivors, with a significant positive correlation between NET concentration and systemic inflammatory markers. This finding underscores the clinical relevance of NETs, even if their definitive role as a therapeutic target remains to be fully established.

### 2.4 NETs and autoimmune nephropathy

The role of NETs in the pathogenesis of autoimmune diseases is an area of intense research. Rather than acting strictly as central drivers in all contexts, NETs may function as biomarkers of disease severity, secondary amplifiers of inflammation, or direct mediators of organ injury, depending on the specific disease state. Emerging evidence suggests their involvement is multifaceted: for instance, nuclear components released by NETs—including DNA, histones, and their citrullinated derivatives—can serve as autoantigens, potentially contributing to the induction of autoantibodies (28). Concurrently, these components may function as DAMPs that activate pattern recognition receptors, such as Toll-like receptors and the NLRP3 inflammasome, which may further promote the release of pro-inflammatory cytokines like IL-1β and trigger a sustained inflammatory cascade. Furthermore, impaired NET clearance, driven by functional defects in DNase1 or interference by anti-NET antibodies, often leads to their accumulation within tissues. This accumulation is frequently associated with exacerbated autoimmune responses and collateral damage to target organs, a mechanism with varying degrees of contribution across different autoimmune conditions, such as systemic lupus erythematosus and rheumatoid arthritis (147).

#### 2.4.1 AKI caused by NETs and ANCA-associated vasculitis

Anti-neutrophil cytoplasmic antibody (ANCA)-associated vasculitis (AAV) is a group of systemic autoimmune diseases characterised by inflammation of small and medium-sized blood vessels. The pathological core of the condition is the abnormal activation of neutrophils mediated by ANCA, leading to damage to the vessel walls and impaired organ function. The condition primarily comprises granulomatous polyangiitis (GPA, where granulomas are present but asthma is absent), microscopic polyangiitis (MPA, where vasculitis is present but there is no evidence of granulomas or asthma) and eosinophilic polyangiitis (EGPA, also known as Churg-Strauss syndrome) (148), which can affect multiple organ systems and commonly manifest as inflammatory lesions in the respiratory tract, kidneys and skin (149, 150).

**Figure 4:**
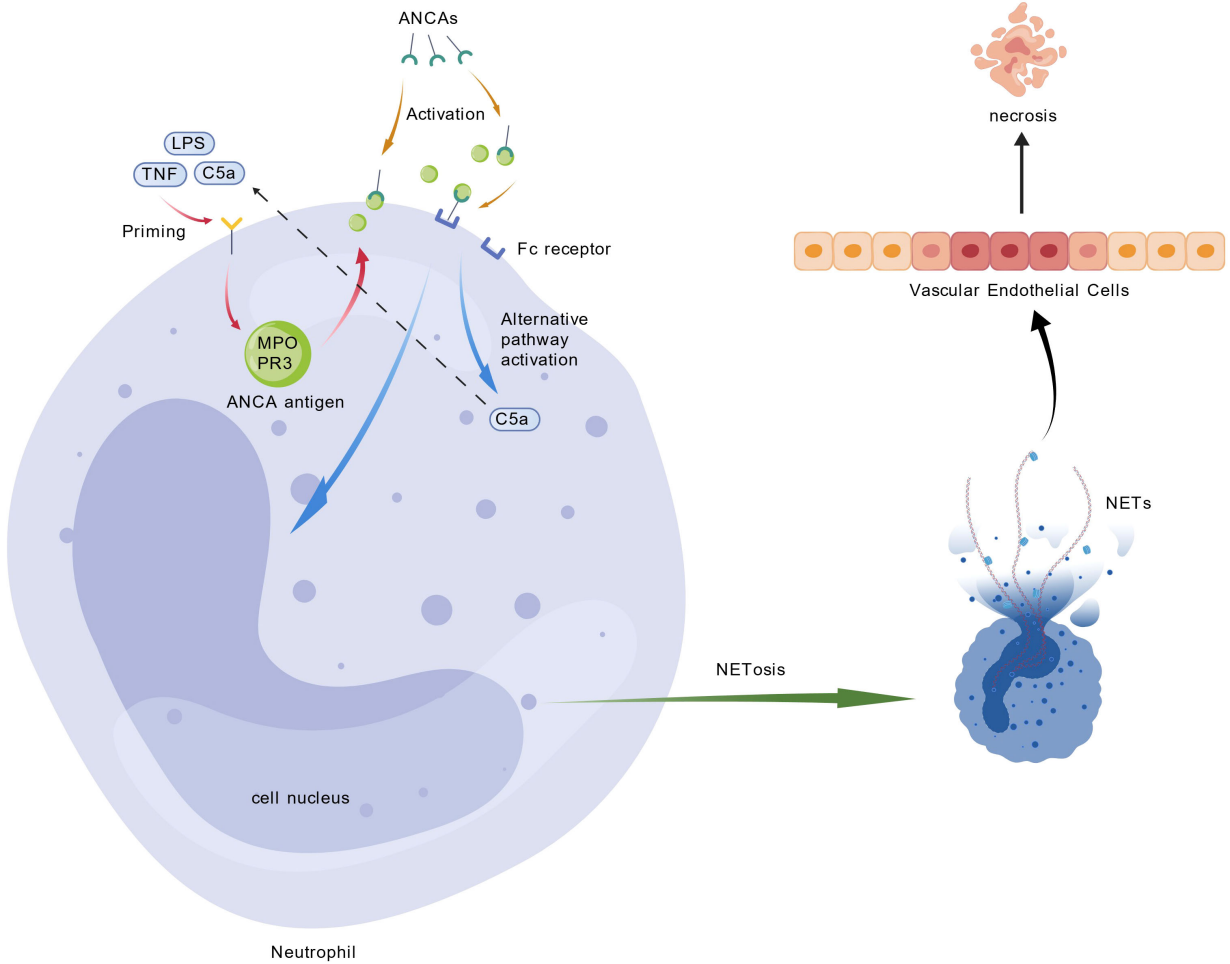
Pathogenesis of AAV. Circulating neutrophils are pre-activated by inflammatory cytokines or by C5a released via the complement pathway, thereby releasing intracellular ANCA-associated antigens into the microenvironment, where they subsequently interact with ANCA. This interaction is mediated either by the cross-linking of ANCA with Fcγ receptors on the neutrophil surface or by the direct binding of the F(ab’) fragment of ANCA to surface antigens, both of which lead to neutrophil activation. ANCA-activated neutrophils release C5a generated via the complement alternative pathway. C5a and ANCA form a vicious cycle, wherein C5a attracts and activates more neutrophils, thereby further activating the complement alternative pathway and producing more C5a. Furthermore, ANCA-activated neutrophils undergo NETosis, releasing NETs into the microenvironment, which in turn leads to apoptosis and necrosis of vascular endothelial cells [(Created with BioGDP.com (41)].

As the central initiators and amplifiers of the pathogenesis, neutrophils are not only the primary target cells for ANCA attack but also the key effector cells responsible for inflammation and tissue damage (151). Under the influence of pro-inflammatory stimuli, such as the cytokine TNF (tumour necrosis factor), bacterial LPS or complement cleavage products such as C5a, circulating neutrophils undergo a critical ‘activation’ process, This activation causes neutrophils to transport their intracellularly stored ANCA-targeted antigens, particularly MPO (152) and PR3 (153), to the cell surface or release them into the microenvironment (154). Subsequently, these exposed target antigens bind to pathogenic anti-neutrophil cytoplasmic autoantibodies (ANCAs) (151) in the circulation; this binding is mediated either by the cross-linking of ANCA-IgG with Fcγ receptors (FcγRs) on the neutrophil surface or by the direct binding of the F(ab’) fragment of ANCAs to surface antigens (155). The activation of p38 MAPK and ERK (151, 156, 157) plays a crucial role in the initiation and activation of neutrophils, subsequently triggering intracellular signalling pathways that lead to the ‘full activation’ of neutrophils. As a direct consequence of neutrophil activation, they undergo intense respiratory bursts, degranulation and, more crucially, the extrusion of NETs, a process known as NETosis. Furthermore, these ANCA-induced activated neutrophils also activate the alternative complement pathway, producing C5a (158, 159); this C5a, in turn, activates further neutrophils (160) and acts as a chemotactic factor to recruit more neutrophils to the site of inflammation, thereby amplifying the inflammatory response and creating a vicious cycle (161) (**Figure 4**).

ANCA-associated vasculitis frequently affects the kidneys, and its most characteristic clinical feature is rapidly progressive renal failure. In clinical diagnosis, this presentation—marked by a rapid rise in serum creatinine and a sharp decline in renal function over a short period—constitutes a severe form of AKI. The primary mechanism underlying this is that ANCA activates neutrophils to attack small blood vessels, leading to glomerular necrosis. Extensive glomerular necrosis and the formation of crescents result in clinical AKI or RPGN (58, 162, 163).NETs play a crucial role in this process; as fibrillar structures, they can adhere to the endothelial cells of glomerular capillaries. This adhesion not only causes direct tissue damage but also physically obstructs microvascular blood flow—forming a three-dimensional scaffold within the vessels that traps circulating platelets and red blood cells like a ‘fishing net’, whilst also actively stimulating thrombotic reactions (79). The direct damage caused by NETs largely stems from the ‘weapons’ they carry, such as NE, MPO and PR3; these proteins possess proteolytic activity or the ability to generate potent oxidants. Interestingly, NETs can also localise these harmful substances within the glomerulus, prolonging the duration of their destructive effects on endothelial cells and the basement membrane, thereby exacerbating the damage. Biopsy analysis of renal specimens from patients with MPO-ANCA-associated glomerulonephritis has detected three key components of NETs in cases of glomerular fibrinoid necrosis and interlobular arterial wall damage: citrullinated histones, MPO and PAD4 were highly co-localised; citrullination marks NET formation, whilst PAD4 promotes histone citrullination, further confirming that NETs may be a key component of the pathological process in ANCA-associated vasculitis (164). As AAV is a relapsing-remitting disease, patients are highly prone to relapse within five years of successful treatment. Without treatment, the one-year mortality rate is approximately 80%, but with current therapies, the five-year mortality rate can be reduced to 25% (165, 166); therefore, active treatment is of paramount importance.

#### 2.4.2 NETs and systemic lupus erythematosus nephritis

LN represents a critical organ-threatening manifestation of SLE, affecting over half of SLE patients and often progressing to varying degrees of renal insufficiency (167). The underlying mechanism involves abnormal immune function in SLE patients, leading to the deposition of immune complexes in the glomerular basement membrane, renal tubulointerstitium and other sites, thereby triggering an immune-inflammatory response and renal damage (168, 169). Neutrophil dysfunction plays a pivotal role in the pathological progression of SLE, and this abnormal activation is closely linked to disease onset and development. Nevertheless, a critical distinction must be drawn between preclinical model findings and the heterogeneous clinical manifestations of LN.

In physiological states, NETs serve as host defense mechanisms to trap and degrade extracellular pathogens. *in vitro* and murine models suggest that NETs undergo pathological reprogramming, potentially serving as persistent drivers of autoimmunity. Specifically, damaged or IFN-α-primed neutrophils—particularly low-density granulocytes (LDGs)—exhibit an increased propensity for NETosis (170, 171). Experimental pathways have been proposed, notably the REDD1-mediated autophagy axis, which may suppress mTORC1 signalling to facilitate NET formation (172). Despite these mechanistic insights, the translational gap remains: whether these specific intra-cellular signaling pathways are the primary drivers of human renal injury or merely secondary phenomena within the inflamed kidney requires further validation. The molecular constituents of NETs—specifically DNA, LL37, and human neutrophil peptides (HNP)—are key immunogenic determinants. The binding of LL37 to endogenous DNA creates stable, nuclease-resistant complexes, shifting ‘self’ DNA from non-immunogenic to potent auto-antigens (78). Autoantibodies in SLE patients bind to this DNA to form immune complexes. These complexes readily deposit in the glomerular basement membrane, mesangial region, or subendothelial space, where they activate the complement system and attract further immune cells, leading to vascular inflammation, organ ischaemia, and tissue necrosis (173, 174). Clinically, this may manifest as proteinuria and elevated serum creatinine levels. While it is hypothesized that these complexes deposit in the glomerular niche to amplify complement-mediated damage, it remains a point of clinical debate whether this deposition is the direct initiator of rapidly progressive glomerulonephritis or a contributing factor to injury exacerbation.

Concurrently, the FcγRIIa (CD32) receptor on the surface of pDCs recognises and binds to these antibody-coated NETs complexes (175). Subsequently, receptor-mediated selective endocytosis transports the DNA-antimicrobial peptide complexes into the intracellular compartments of pDCs. Upon entry into the cell, the endogenous DNA within the complexes is recognised by Toll-like receptor 9 (TLR9, which recognises endogenous DNA) (176), inducing a massive transcriptional response in pDCs, which in turn secrete large amounts of IFN-α (type I interferon) to trigger an antiviral immune response. Clinically, this often manifests as fatigue, joint pain, and myalgia—symptoms very similar to those of a ‘severe cold’ or ‘flu’ (177). This response induces the release of pro-apoptotic factors, leading to widespread apoptosis or necrosis of granular cells (such as podocytes or endothelial cells), which in turn causes the glomerular filtration barrier to collapse within a short period. Furthermore, IFN-α can upregulate BLyS (B-lymphocyte stimulator, also known as BAFF) to drive abnormal B-cell activation (178) and lower the activation threshold of the B-cell receptor (BCR) (179), whilst ‘priming’ circulating neutrophils (180), promoting their differentiation and the production of additional autoantibodies against DNA, LL37 and HNP (78). This ‘self-amplifying’ process means that inflammation can escalate dramatically within a very short period of time. The rapid progression from initial mild inflammation to severe, diffuse proliferative glomerulonephritis (such as type IV LN) is often clinically manifested as rapidly progressive glomerulonephritis. Previously, it was thought that the DNA within NETs consisted primarily of nuclear DNA; however, research by Haiting Wang et al. has revealed that NETs are rich in mitochondrial DNA (mtDNA), which is more pathogenic and has a greater capacity to induce interferon production than conventional nuclear dsDNA (181). Interestingly, the correlation between anti-mtDNA antibody levels and renal activity indices is even stronger than that of the clinically commonly used anti-dsDNA antibodies, offering additional diagnostic options for SLE.

Against the pathophysiological backdrop of lupus nephritis, NETs should not be simply regarded as a single pathogenic factor. Instead, they can be recognized as biomarkers of inflammatory severity, amplifiers of secondary injury and potential direct mediators (182). Although NET-derived autoantigens may facilitate immune complex deposition and subsequent complement activation, the precise causal role of NETs in the progression of acute kidney injury remains an area of active investigation. Current scientific evidence reveals a notable discrepancy: ex vivo studies demonstrate that neutrophils from LN patients do not exhibit an inherent hyper-capacity for NETosis, suggesting that the observed accumulation of circulating NET remnants may be primarily attributed to impaired DNase-mediated clearance rather than primary overproduction. Consequently, a more nuanced perspective is required to distinguish whether NETs actively drive renal deterioration or serve as reflective markers of a worsening inflammatory microenvironment, thereby ensuring a scientifically rigorous assessment of their role across diverse autoimmune and inflammatory contexts.

### 2.5 Testing method

The identification of NETs relies primarily on a combined assessment of quantitative circulating biomarkers and tissue microimaging (183), and there is no single ‘gold standard’ method (184, 185) (**Table 2**).

**Table 2:** Methods for detecting NETs.

Method	Advantages	Limitations
ELISA (186)	Simple to operate and suitable for routine quantitative analysis of large numbers of samples	Lack of specificity; there is controversy regarding standardisation
Immunofluorescence Microscopy(IFM) (187, 188)	Intuitive, morphological evidence	Time-consuming, highly subjective, and unsuitable for rapid screening
Electron microscope (183)	High resolution, for viewing minute details	Extremely complex, expensive and prone to artefacts
Conventional flow cytometry (189, 190)	Fast and objective	Difficult to distinguish the mechanism of death
Optimized flow cytometr (191, 192)	Objective, highly specific (double-labelled), capable of distinguishing NETs from apoptosis/necrosis	Requires specialist equipment and a basic understanding of experimental design

Currently, the most commonly used method involves measuring levels of characteristic markers in plasma or urine—such as MPO, cfDNA, Cit-H3 and MPO-DNA complexes—using an enzyme-linked immunosorbent assay (ELISA) to assess the extent of NET formation and the degree of tissue damage in the body (188, 193–195). MPO-DNA complexes suffer from low specificity, as MPO can be released via classic degranulation pathways independently of NET formation (186). Similarly, the diagnostic value of Cit-H3 is limited by two factors: firstly, the heterogeneity of the NET cell death pathway, such as activation mechanisms that do not rely on PAD4; and secondly, the susceptibility of the N-terminal antigenic epitope of histone H3 to proteolytic cleavage by NE, resulting in the instability of the target antigenic epitope. More challenging still is the fact that currently, in-house ELISA protocols vary widely across laboratories and lack standardised inter-laboratory protocols, further hindering the clinical implementation of such biomarkers. To address this bottleneck, Wargnies et al. proposed H3.1-nucleosomes as a novel quantitative marker for NETs. Compared to free DNA, which is susceptible to enzymatic degradation, or histones with highly unstable post-translational modifications, H3.1-nucleosomes, as the basic structural units of chromatin, possess greater structural stability (184). Experiments using immunofluorescence and immunoprecipitation assays in *in vitro* models (PMA/CI-induced HL-60 cells and primary neutrophils) confirmed that H3.1-nucleosomes exhibit close spatial association and physical interactions with key components of NETs, validating their biological plausibility as representatives of the structural entity of NETs. However, current clinical validation is limited to retrospective cohort studies; its prognostic value in actual clinical decision-making and the results of independent external validation still require assessment through long-term prospective studies, and it cannot yet be regarded as the definitive solution to the controversy surrounding NETs detection (196).

### 2.6 Summary

NETs play a significant role in the pathological progression of AKI. The extracellular histones released by NETs exert direct cytotoxic effects on renal tubular epithelial cells, sufficient to induce extensive cellular necrosis and apoptosis, particularly during the early triggering phase of acute tubular necrosis (ATN). Concurrently, as potent DAMPs, NETs can continuously amplify the release of pro-inflammatory cytokines by activating the TLR2/TLR4 signalling pathways and the NLRP3 inflammasome, thereby inducing the further release of neutrophils. Furthermore, the fibrillar network formed by NETs readily causes renal microvascular thrombosis and luminal obstruction, exacerbating renal ischaemia-hypoxia and haemodynamic disturbances. This mechanism of injury, jointly mediated by direct cell lysis, immune-inflammatory cascades and microcirculatory dysfunction, accelerates the pathological progression of renal function from primary injury to rapid failure.

## 3 Anti-NETs therapy

Therapeutic strategies for NETs primarily focus on three aspects: inhibiting their formation and release, degrading existing structures, and clearing them via *in vitro* methods (197) (**Table 3**).

**Table 3:** Treatment plan.

Treatment categories	Representative drugs/methods	Mechanism of action	Limitations and challenges
PAD4 inhibitor	Cl-amidine (198, 199)	Inhibiting PAD4 blocks histone citrullination and chromatin decondensation, thereby reducing NET formation at its source.	Members of the PAD family are highly conserved, making it difficult to achieve selectivity for a single target; Cl-amidine requires complex delivery systems.
Lactoferrin (Lf)	Lf (200)	Through the interaction between positive charges and the negative charges on DNA, chromatin is concentrated near the cell membrane, preventing its diffusion.	There is currently insufficient evidence regarding the route of administration, timing of administration and the efficacy of the drug in the complex human environment.
DNases	DNase I (201)	Hydrolysis of circulating and tissue-bound free DNA (DAMPs) and NET components; RNase III specifically cleaves dsRNA.	The therapeutic efficacy is highly dependent on the pathological model (e.g. it is primarily effective in ischaemic mechanisms); clinical translation is challenging.
Non-invasive treatment methods	PMX-DHP (202)	Circulating NETs are removed via physical filtration, charge adsorption and hydrophobic interactions; TPE can supplement endogenous nucleases.	Synthetic materials may trigger biocompatibility reactions, leading to new oxidative stress and NETs; therefore, the dialysis regimen needs to be optimised.
promising drugs	Aspirin (203)	By inhibiting the phosphorylation of NF-κB p65, it remains effective in suppressing NET formation even under conditions of acidosis or heat stress.	It increases the risk of bacteraemia, and long-term use may cause side effects such as gastric ulcers and gastrointestinal bleeding.
promising drugs	Metformin (181, 204)	Inhibits PKC-βII and NOX, thereby preventing ROS bursts and PAD4-mediated histone citrullination.	As it is excreted via the kidneys, it tends to accumulate during AKI, leading to lactic acidosis; it is therefore an absolute clinical contraindication during the acute phase of AKI.

### 3.1 PAD4 inhibitor

PAD4 plays an indispensable role in mediating histone citrullination and the subsequent chromatin decondensation process. It has emerged as a key therapeutic target for regulating and interfering with the formation of NETs. Based on their mechanisms of action, these inhibitors are classified into two main categories: irreversible and reversible (48). Irreversible inhibitors, such as Cl-amidine, primarily act by forming a covalent bond between their halogenated amide group and the Cys645 residue within the active site of PAD4, thereby blocking the enzyme’s catalytic activity and significantly inhibiting the formation of NET-like structures. Although Cl-amidine has been validated in animal models as having significant therapeutic value in modulating the pathological progression of AKI and improving the prognosis of multi-organ dysfunction (205, 206), PAD inhibitors represented by Cl-amidine are highly dependent on advanced drug delivery systems (such as P(3HB) microsphere encapsulation technology) and sensitive bio-monitoring methods, which poses a challenge for clinical translation. In contrast, reversible inhibitors such as GSK199 exhibit greater chemical structural diversity; they exert their inhibitory effects by occupying the U-shaped tunnel of the PAD4 active site or by interacting with active site residues via guanidinium groups. In mouse models (207, 208), GSK484 blocks the formation of NETs by specifically inhibiting PAD4 activity, effectively alleviating renal inflammation, improving blood perfusion and mitigating associated AKI. Although PAD4 inhibitors have demonstrated significant potential in preclinical studies for alleviating autoimmune inflammation and reducing thrombogenesis, no drug has yet entered clinical use. This is primarily because members of the PAD family are highly conserved evolutionarily, exhibiting particularly high structural similarity in the active site region; this poses a significant challenge to the design of specific drugs that target only PAD4 whilst avoiding interference with other isoforms possessing normal physiological functions (48, 209, 210).

### 3.2 Lactoferein

Lactoferrin (Lf) is primarily found in breast milk, tears and the granules of neutrophils. Researchers have discovered that lactoferrin, previously thought to be merely a component of NETs, is in fact a natural inhibitor of NET release. Research (200) has shown that lactoferrin utilises its positively charged amino acid sequence to form physical charge interactions with negatively charged DNA, thereby causing chromatin fibres to aggregate and bind near the cell membrane. Under conditions that do not interfere with activation processes such as ROS production, histone citrullination or elastase nuclear entry, this significantly inhibits the excessive dissemination of NETs at inflammatory sites and within the bloodstream. A 12-amino acid engineered short peptide in lactoferrin, named M10Hse (Me), has successfully condensed the function of the biomolecule into a medium-molecular-weight drug with greater clinical utility. In a mouse model of rhabdomyolysis-induced acute kidney injury (RIAKI), Me was shown to alleviate the toxicity of NETs to renal tubules by specifically inhibiting their release (211). However, the selection of administration routes and timing, as well as the validation of efficacy in the more complex human environment, remain challenges for its clinical translation.

### 3.3 DNases

Deoxyribonucleases (DNases) are a class of enzymes that play a central role in maintaining human homeostasis; their primary physiological function is to hydrolyse deoxyribonucleic acid molecules in the circulatory system and within organ tissues, thereby preventing extracellular DNA from inducing excessive inflammatory responses as DAMPs. Biologically, DNases are classified into the DNase I family, which is divalent metal ion-dependent and functions in a neutral environment, and the DNase II family, which acts in the acidic environment of lysosomes (212). Among these, serum DNase I has been demonstrated to be a key factor in the degradation of NETs (201). In animal studies of AKI, it has been demonstrated that exogenous supplementation with DNase I accelerates the clearance of extracellular free DNA, significantly improves renal perfusion and alleviates tissue hypoxia, thereby reducing the levels of apoptosis and necrosis in renal tubular epithelial cells and lowering functional indicators such as plasma creatinine and blood urea nitrogen (20, 213–215). However, the role of DNase I in AKI is highly dependent on the pathological model; although it has clear protective value in ischaemic AKI, this has become a major challenge for its translation into clinical practice. In recent studies, RNase III, a ribonuclease that specifically degrades double-stranded RNA (dsRNA), differs from traditional DNase I in that it can more precisely clear pathogenic dsRNA components from NETs, and may thus represent a novel therapeutic strategy for blocking NET-induced damage to renal tubular epithelial cells (216).

### 3.4 Non-invasive treatment methods

Literature studies have shown that haemoperfusion using a polyoxymethylene-immobilised polyoxymethylene-DHP (PMX-DHP) filter cartridge significantly reduces the concentrations of key biomarkers reflecting NETs levels in plasma—namely MPO-DNA, NE-DNA and cell-free DNA (cf-DNA)—in both *in vitro* circuit simulations and *in vivo* clinical applications in patients with septic shock. This finding strongly demonstrates that the *in vitro* adsorption and removal of circulating NET components via a haemoperfusion system represents a highly promising therapeutic strategy (202). As extracorporeal circulation circuits and dialysis membranes are non-natural, artificial materials, contact between blood and these surfaces can trigger biocompatibility reactions, directly activating neutrophils and inducing the production of ROS. This physical contact and oxidative stress can trigger the release of additional NET components from neutrophils, including DNA, Cit-H3 and NE (217). Therefore, in the *in vitro* treatment of AKI, the key to improving clinical outcomes may lie in selecting high-flux or convection-dominated dialysis modes (218), thereby reducing the stimulatory effect of inflammatory mediators at the ‘source’ and indirectly alleviating the persistent immune damage caused by NETs to the kidneys and systemic vasculature. Alternatively, could we enhance the physical interception of macromolecular nucleic acid-protein complexes by developing high-retention dialysis membranes, or utilise membrane surface charge modification techniques (such as the positively charged layer on oXiris membranes) and high-efficiency adsorption resins (such as CytoSorb) to capture charged DNA fragments and histones via electrostatic attraction and hydrophobic interactions, whilst combining this with therapeutic plasma exchange (TPE) to replenish endogenous nucleases depleted by the disease, thereby promoting NET degradation and achieving a multidimensional clearance effect ranging from physical filtration and chemical adsorption to biodegradation (219).

### 3.5 Promising drugs

A study by Lapponi et al. demonstrated that aspirin (ASA) significantly reduces the formation of NETs by inhibiting the phosphorylation of the NF-κB p65 subunit, and that this effect is independent of the ERK pathway and apoptosis. Even under stressful conditions such as acidosis (pH 6.5) or hyperthermia (42 °C), ASA remains effective in inhibiting NETosis (220).However, animal studies suggest that whilst the aspirin-mediated inhibition of NETs alleviates local inflammatory responses, it may increase the risk of bacteraemia. Furthermore, as aspirin possesses anticoagulant properties, long-term use may lead to complications such as gastric ulcers and gastrointestinal haemorrhage. Consequently, further research is required to assess the balance between its therapeutic potential and the associated risks of infection. Metformin, currently the only drug with dual effects of glucose-lowering and NETosis inhibition, directly inhibits PKC-βII membrane translocation and NOX activity, thereby blocking hyperglycaemia-induced ROS bursts and PAD4-mediated histone citrullination, thus significantly suppressing the NETosis process (221). However, its clinical application in the treatment of AKI remains fraught with challenges: as the drug is renally excreted, the rapid decline in renal function associated with AKI leads to rapid drug accumulation, thereby inducing metformin-associated lactic acidosis, which carries an extremely high mortality rate. Consequently, current clinical guidelines classify its use as an absolute contraindication during the acute phase of AKI and in cases of haemodynamic instability. Furthermore, the protective effects of metformin in inhibiting NETosis are currently based largely on early intervention in animal studies; there remains a lack of sufficient evidence-based medical data to determine whether the therapeutic benefits can outweigh the potential risks of metabolic disturbances in the complex clinical setting of established AKI.

Currently, targeting NETs is rapidly emerging as a therapeutic approach for a variety of diseases. However, different compounds used to inhibit or eliminate NETs may have other adverse effects on the immune system; consequently, further research is needed to elucidate their mechanisms of action so that we can weigh up the benefits against the risks. The management of NETs may require a combination therapy, combining conventional treatments (such as fluid therapy, antibiotics and antiviral drugs) with NET-targeting drugs, in order to achieve better therapeutic outcomes.

## 4 Summary and outlook

In summary, NETs represent a pivotal but complex mediator in AKI, balancing antimicrobial defense with the promotion of sterile inflammation and renal damage. While targeting NET-related pathways offers promising therapeutic potential, current evidence often leans heavily toward their deleterious roles, leaving their homeostatic and protective functions under-explored. A primary limitation in the field remains the heavy reliance on animal models, which may not fully replicate the temporal kinetics and heterogeneity of clinical AKI. Furthermore, significant clinical uncertainty persists due to the lack of validated, real-time biomarkers to monitor NET levels and the inherent risk that broad inhibition might compromise systemic host immunity and pathogen clearance.

Future research must therefore move beyond simple inhibition toward a more nuanced and critical approach, prioritizing the distinction between pathological and physiological NETosis. A key directive involves the development of kidney-targeted delivery systems to enhance therapeutic specificity and minimize systemic off-target effects. Additionally, longitudinal studies in human cohorts are essential to define the optimal therapeutic window for intervention. By addressing these methodological gaps and deciphering the context-specific roles of individual NET components, we can transition from generalized suppression to personalized, precision strategies that mitigate renal injury while preserving essential immune functions.
